# Oral Frailty and Physical Frailty Associated With Sleep Quality in Community‐Dwelling Adults: A Cross‐Sectional Study

**DOI:** 10.1111/joor.70152

**Published:** 2026-01-24

**Authors:** Pei‐Chen Lin, Ai‐Hua Chang, Shin‐Ru Liao, Koichiro Matsuo, Yuji Kabasawa, Ju‐Hui Wu, Pei‐Chao Lin, Hsiao‐Ling Huang

**Affiliations:** ^1^ Department of Oral Hygiene, College of Dental Medicine Kaohsiung Medical University Kaohsiung Taiwan; ^2^ Department of Dentistry Kaohsiung Medical University Hospital Kaohsiung Taiwan; ^3^ Department of Medical Research Kaohsiung Medical University Hospital, Kaohsiung Medical University Kaohsiung Taiwan; ^4^ School of Dentistry, College of Dental Medicine Kaohsiung Medical University Kaohsiung Taiwan; ^5^ Department of Oral Care for Systemic Health Support, Graduate School of Medical and Dental Sciences Institute of Science Tokyo Tokyo Japan; ^6^ Department of Oral Health Sciences for Community Welfare, Graduate School of Medical and Dental Sciences Institute of Science Tokyo Tokyo Japan; ^7^ College of Nursing Kaohsiung Medical University Kaohsiung Taiwan; ^8^ Center for Long‐Term Care Research Kaohsiung Medical University Kaohsiung Taiwan

**Keywords:** dry mouth, OF, oral, oral frailty, rehabilitation, sleep quality

## Abstract

**Background:**

Oral frailty and physical frailty are linked to adverse health outcomes in older adults, but their relationship with sleep quality is not well understood. This study examined the association between oral frailty and sleep quality, and the combined effects of oral and physical frailty on sleep in community‐dwelling older adults.

**Methods:**

A cross‐sectional study was conducted in Kaohsiung City, Taiwan (2018–2019), including 1180 adults aged ≥ 65 years selected by multistage stratified cluster sampling. Data were collected through questionnaires and dental exams. Sleep quality was measured by the Pittsburgh Sleep Quality Index. Oral frailty was assessed using indicators such as oral diadochokinesis, swallowing difficulty, masticatory performance, dry mouth, tongue coating, and number of natural teeth. Oral health–related quality of life (OHRQoL) was evaluated with the GOHAI. Logistic regression identified factors associated with poor sleep quality.

**Results:**

Swallowing difficulty (AOR = 2.69) and dry mouth (AOR = 2.06) were significantly linked to poor sleep quality. A dose–response relationship was observed across stages of swallowing difficulty, with both suspected and confirmed cases reporting significantly poorer sleep quality compared to individuals without swallowing issues (p for trend < 0.001). Higher OHRQoL scores were linked to better sleep quality (AOR = 0.97). Additionally, the combined presence of oral frailty and physical frailty was significantly associated with poor sleep quality (AOR = 2.71).

**Conclusion:**

Oral and physical frailty are significantly associated with sleep quality in older adults. Interventions targeting swallowing difficulty, dry mouth, and physical frailty may improve sleep quality in this population.

## Graphical abstract

In community‐dwelling adults aged ≥ 65 years, swallowing difficulty and dry mouth were significantly associated with poor sleep quality. The combined presence of oral frailty and physical frailty further increased the risk of poor sleep quality.

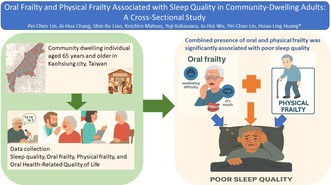

## 1 Introduction

Globally, sleep disorders are common among community‐dwelling older adults. The most prevalent sleep problems in this population include obstructive sleep apnoea (46.0%), poor sleep quality (40.0%), other sleep disturbances (37.0%), insomnia (29.0%) and excessive daytime sleepiness (19.0%) [1]. Poor sleep quality is significantly associated with physical frailty in older adults [2]. Compared to their robust counterparts, frail older adults tend to have more comorbidities and poorer sleep quality [3]. Longitudinal studies have shown that poor sleep quality is linked to increased physical frailty over time. Changes in frailty may mediate the relationship between overall sleep quality and various sleep parameters, including subjective sleep quality, sleep duration, sleep efficiency, and daytime dysfunction [4]. Furthermore, the coexistence of poor sleep and physical frailty can lead to adverse health outcomes in older adults, such as an increased risk of falls, dementia, depression, heart failure, reduced quality of life and higher mortality rates [5, 6, 7].

Declines in oral function have been shown to progress stepwise, in parallel with the deterioration of systemic function. A conceptual framework has been proposed to categorise this process into four stages: a healthy state, oral frailty (OF), oral hypofunction, and oral dysfunction. Notably, the stages of oral frailty and oral hypofunction are considered reversible through appropriate interventions tailored to each level. To date, however, there is no universally accepted definition of oral frailty, and the conceptual boundaries between OF and oral hypofunction continue to be refined as research in this area evolves. Tanaka et al. (2018) [8] proposed a six‐item assessment for oral frailty, which included the number of natural teeth, chewing ability, articulatory oral motor skills for the ‘ta’ sound, tongue pressure, subjective difficulty in eating tough foods, and subjective difficulty in swallowing. Oral frailty (OF) was defined as the coexistence of poor status in three or more of these six measures. Subsequently, in 2024, international experts reached a consensus on the operational definition of oral frailty, suggesting that it should encompass the following components: (1) difficulty eating hard or tough foods, (2) inability to chew all types of foods, (3) decreased ability to swallow solid foods, (4) decreased ability to swallow liquids, (5) overall poor swallowing function, (6) impaired tongue movement, (7) speech or phonatory disorders, and (8) hyposalivation or xerostomia [9].

Research has indicated that the prevalence of oral frailty among older adults varies from 8.1% to 53.2%, with pre‐frailty rates ranging from 33.7% to 75.6% [10]. According to a survey conducted in Japan, 16% and 50% of individuals were identified as having oral frailty and pre‐OF, respectively [8]. In Taiwan, the prevalence rates are higher, with 20.7% for oral frailty and 57.2% for pre‐OF. Notably, among individuals aged 75 and older, the prevalence of oral frailty is approximately twice as high as in those under 75 years of age (30.2% vs. 15.0%) [11]. In recent years, research has increasingly focused on the relationship between OF and overall health in older adults, revealing that those with OF are more likely to develop physical frailty [12]. A meta‐analysis concluded that deterioration in oral health status (52%), particularly having few remaining teeth (29%), was most frequently associated with physical frailty. Other less common but still significant factors included reduced oral motor skills (27%)—particularly masticatory function, oral diadochokinesis, and occlusal force—as well as disorders related to chewing, swallowing, and saliva production (20%), with chewing difficulties being the most notable among them [10]. Key factors influencing OF in community‐dwelling older adults include demographic characteristics (such as age, sex and educational level), the number of dentures, poor oral health status, dry mouth, subjective chewing difficulty, degree and stage of physical frailty, and sleep quality [13].

There is a limited body of literature examining the correlation between OF and sleep quality. To date, only one study [14] has primarily investigated the impact of sleep disorders on oral health, focusing on indicators such as dental plaque and calculus accumulation, gingival inflammation, and masticatory function. However, OF in older adults involves broader functional impairments, including difficulties with chewing, swallowing and dry mouth. To address this gap, the present study is the first to investigate the relationship between both oral and physical frailty and sleep quality in community‐dwelling older adults. The primary aim was to explore the association between OF and sleep quality, with additional analysis of the combined effects of oral and physical frailty on sleep quality outcomes.

## 2 Materials and Methods

### 2.1 Study Design and Participants

This cross‐sectional study included community‐dwelling participants aged 65 years and older residing in Kaohsiung, Taiwan, from May 2018 to January 2019. This study employed a multistage stratified cluster sampling method to select individuals from an older adult population. Kaohsiung City comprises 39 districts categorised into urban, rural, and mountainous areas. In the first stage of sampling, 25 districts were randomly chosen from 39 districts using a probability proportional to size method. In the second stage, community care centres within these 25 districts were randomly selected. Finally, adults aged 65 years and older were recruited from each selected community care center, resulting in a total of 1180 enrolled participants.

Participants were considered invalid samples if the sleep quality information from Pittsburgh Sleep Quality Index was missing (response rate: 88%). Additional, samples were excluded if participants self‐reported any of the following: (a) mental disorders, (b) communication disorders, (c) moderate or severe cognitive impairment, as determined by a short portable mental status questionnaire (SPMSQ) [15], (d) high‐to‐total dependence in Activities of Daily Living (ADL) [16], or (e) moderate‐to‐severe depression, as assessed using the Geriatric Depression Scale (GDS) [17]. The final analysis included a total of 935 participants.

The sample size was estimated using G*Power version 3.1.9.4 [18]. Post hoc power was computed using the effect size, calculated as the ratio of variance between sleep quality and sample size and the α level was 0.05. The power was greater than 0.8, indicating that the sample size was sufficiently large.

### 2.2 Oral Examination

Oral examinations were conducted by seven dentists according to World Health Organization criteria [19]. The interrater agreement for tooth decay using the kappa coefficient was 0.77. Kendall's W coefficient for the plaque index was 0.87 for interrater agreement. All oral health indices included the number of natural teeth, and mobility was recorded by a dentist using a CPI explorer and dental mirror.

### 2.3 Instrument

A structured questionnaire [20] was used to collect demographic information (i.e., age, sex, and education level), physical condition (i.e., comorbidities, sarcopenia, and physical frailty), OF (i.e., oral diadochokinesis, swallowing difficulty, masticatory performance, dry mouth, tongue coating and number of natural teeth) and sleep quality. Items were reviewed by a panel of experts to assess content validity, resulting in an index ranging from 0.89 to 1.00, which was considered adequate. To ensure that the study participants understood the content, the questionnaires were pilot tested with 30 older adults.

### 2.4 Dependent Variable

#### 2.4.1 Sleep Quality

Sleep quality was assessed using the Pittsburgh Sleep Quality Index (PSQI), which evaluates self‐reported sleep patterns over the past month. The index consists of seven components: subjective sleep quality, sleep onset latency, total sleep duration, sleep efficiency, sleep disturbances, use of sleep medication and daytime dysfunction and is measured using 19 items. The total score ranged from 0 to 21, with higher scores indicating poorer overall sleep quality. The cut‐off score was 5, and poor sleep quality was > 5 [21]. The Chinese version of the PSQI cutoff score of 5 has a sensitivity of 98% and a specificity of 55% [22].

### 2.5 Independent Variable

#### 2.5.1 Physical Condition

##### 2.5.1.1 Sarcopenia

The Sarcopenia Functional Questionnaire (SARC‐F) exhibits high specificity (94%–99%) and serves as an effective option for screening sarcopenia within the community [23]. It comprises five components: strength, assistance with walking, rising from a chair, climbing stairs and occurrence of falls. Total scores ranged from 0 to 10, with each component contributing between 0 and 2 points. A score of 4 or higher is indicative of sarcopenia.

#### 2.5.2 Physical Frailty

The Study of Osteoporotic Fracture Index (SOF index) is considered as effective as the frailty criteria in predicting adverse health outcomes and is easier to use [24]. The SOF index consists of three components: weight loss, chair stand and energy level, with scores ranging from 0 to 3. Older adults receive one point for each of the following: experiencing a weight loss of 5% or more in the past year, being unable to complete five consecutive chair rises and reporting low or no energy. The total scores categorised the individuals as follows: scores of 2 or 3 indicated frailty, a score of 1 reflected pre‐frailty and a score of 0 indicated robustness.

#### 2.5.3 Comorbidities

Comorbidities were assessed through the self‐report of one or more chronic diseases, including hypertension, diabetes and stroke.

##### 2.5.3.1 Oral Frailty

OF was defined as the presence of at least three of the following indicators: oral diadochokinesis, swallowing difficulty, masticatory performance, dry mouth, tongue coating and number of natural teeth. Pre‐oral frailty (Pre‐OF) was defined as the presence of one to two indicators [11].

#### 2.5.4 Oral Hygiene

Tongue coating was evaluated as an indicator of oral hygiene using the Winkel Tongue Coating Index [25]. The tongue surface was divided into six regions—three anterior and three posterior—and each area was scored as 0 (no coating), 1 (light coating), or 2 (severe coating), yielding a total score ranging from 0 to 12.

#### 2.5.5 Oral Diadochokinesis

DDK was assessed by evaluating articulatory oral motor skills at specific sites, including the tongue tip [26]. Older adults were instructed to repeatedly pronounce the monosyllable ‘TA’ as quickly as possible for 5 s, with the number of syllables pronounced per second being recorded. The oral DDK was calculated based on the articulation count per second and categorised as 6 times or less per second.

#### 2.5.6 Dysphagia

Dysphagia was assessed using the Ohkuma questionnaire, which includes 15 items [27]. Some of the questions were ‘Do you ever have difficulty when you swallow?’, ‘Does it take you longer to eat a meal than before?’, and ‘Do you ever drop food from your mouth?’. Respondents can answer with ‘obviously’ (many times), ‘slightly’ (sometimes), or ‘no’ (never). Individuals with at least one severe symptom were classified as having dysphagia. The questionnaire demonstrated strong internal consistency with a Cronbach's alpha of 0.85.

#### 2.5.7 Masticatory Performance

Masticatory performance was assessed using colour‐changing chewing gum (Gum XYLITOL, Lotte, Tokyo, Japan). This gum contains xylitol, citric acid and red, yellow and blue dyes. As the participants chewed, the yellow and blue dyes were released into the saliva and citric acid caused the gum to turn red. Participants were instructed to chew the gum for 2 min. An observer then evaluated the colour of the gum in a semi‐quantitative manner using a colour chart with five gradations ranging from 1 (very poor) to 5 (very good). For statistical analysis, we categorised masticatory performance into three groups: scores of 1–3 were considered poor, a score of 4 was moderate and a score of 5 was deemed good. The validity and reliability of using colour‐changing chewing gum to evaluate masticatory performance have been previously verified [28].

### 2.6 Dry Mouth

A condensed version of the Xerostomia Inventory was used to assess and categorise mouth dryness [29]. The inventory comprises five statements: ‘My mouth feels dry,’ ‘I have difficulty eating dry foods,’ and ‘My lips feel dry.’ Each statement is rated on a scale of 1 (never), 2 (occasionally), or 3 (often). Total scores ranged from 5 to 15 points, with higher scores indicating greater mouth dryness. Respondents who achieved a total score of 10 points or higher represented the upper 50% of xerostomia scores. The internal consistency of the inventory was measured using Cronbach's alpha, which was calculated as 0.70.

#### 2.6.1 Oral Health‐Related Quality of Life

Oral Health‐Related Quality of Life (OHRQoL) was assessed using the Chinese version of the Geriatric Oral Health Assessment Index (GOHAI), which consists of 12 items divided into three domains: physical function (eating, speech, and swallowing), psychosocial function (concerns about oral health, dissatisfaction with appearance, self‐consciousness related to oral health, and avoidance of social interaction due to oral problems), and pain or discomfort (use of medication to alleviate oral pain or discomfort) [30]. Each item on the GOHAI was rated on a 5‐point Likert scale, where 1 indicated “always” and 5 indicated “never.” Total scores ranged from 12 to 60 points, with higher scores reflecting a more favorable OHRQoL. The internal consistency of the scales was indicated by a Cronbach's alpha of 0.75.

#### 2.6.2 Data Collection

Data were collected through face‐to‐face interviews conducted by well‐trained interviewers following a standardised protocol. The data collection process consisted of three distinct steps of approximately one hour each. Initially, the dentist performed a dental examination and documented the dental status for approximately 10 min. Subsequently, the interviewer administered a structured questionnaire that required approximately 30–45 min to complete. Finally, the research personnel evaluated the oral DDK rate within 30 s.

### 2.7 Statistical Analysis

Statistical analyses were conducted using SPSS (version 20.0; IBM SPSS Statistics for Windows; IBM Corp). The older adults were categorised into two groups based on sleep quality. Several statistical analyses were performed to examine the relationship between sleep quality and various oral and physical health factors. Chi‐square tests were used to assess the associations between categorical variables (e.g., sex, OF, edentulism, and mobility level) and sleep quality. For variables with ordinal categories (such as frailty levels or GOHAI scores), the *p*‐value for the trend was calculated to identify statistically significant trends. Odds ratio (OR) and 95% confidence intervals were computed to evaluate the strength of association between key oral conditions and sleep quality. After adjusting for age, sex, and educational level, multiple logistic regression models were conducted to determine the independent effects of oral function variables on sleep quality. Statistical significance was set at *p* < 0.05 for all tests.

## 3 Results

Baseline information for the older adult participants, categorised by age is presented in Table 1. A total of 29.4% were male and 70.6% female patients, with a mean age of 73.8 ± 6.3 years. Significant differences were observed between the two age groups with respect to educational level (*p* < 0.001), history of hypertension (*p* = 0.002), and history of stroke (*p* = 0.012).

**TABLE 1:** Baseline information of participants by age (*N* = 935).

Variable	Total	< 75‐year‐old, *N* = 588	≥ 75‐year‐old, *N* = 347	*p*
	*N* (%)	*N* (%)	*N* (%)	
Age (mean ± SD)	73.8 ± 6.3	69.72 ± 2.8	80.62 ± 4.3	
Gender				
Women	660 (70.6)	407 (69.2)	253 (72.9)	0.231
Men	275 (29.4)	181 (30.8)	94 (27.1)	
Education level				
Illiterate/elementary school	409 (43.7)	188 (32.0)	221 (63.7)	< 0.001
Junior high/high school	323 (34.5)	234 (39.8)	89 (25.7)	
Above technical school/college	203 (21.7)	166 (28.2)	37 (10.7)	
BMI (mean ± SD)	24.24 ± 3.5	24.40 ± 3.5	23.95 ± 3.5	0.056
Comorbidity				
DM	187 (20.0)	109 (18.5)	78 (22.5)	0.146
HT	424 (45.3)	244 (41.5)	180 (51.9)	0.002
Stroke	37 (4.0)	16 (2.7)	21 (6.1)	0.012

Abbreviations: BMI, body mass index; DM, diabetes mellitus; HT, hypertension; SD, standard deviation.

The relationships between sleep quality and various oral and physical health conditions are presented in Table 2. OF was associated with poor sleep quality [Crude OR (COR) = 2.30, 95% CI: 1.24–4.40]. A dose–response trend in sleep quality was observed across frailty stages, from robust to pre‐OF and OF (*p* for trend < 0.001). Regarding chewing function variables, being edentulous and having limited mobility (Level III) were both significantly associated with sleep quality, with COR of 0.59 and 1.96, respectively. Notably, both suspected swallowing difficulty (COR = 2.19, 95% CI: 1.63–2.98) and confirmed swallowing difficulty (COR = 3.26, 95% CI: 2.06–5.21) were significantly associated with poor sleep quality. A dose–response relationship was observed across stages of swallowing difficulty, with both suspected and confirmed difficulty groups exhibiting significantly poorer sleep quality compared to individuals without swallowing issues (*p* for trend < 0.001). Dry mouth was significantly associated with poor sleep quality (COR = 2.82, 95% CI: 1.75–4.50). Furthermore, GOHAI scores differed significantly by sleep quality (*p* < 0.001), indicating that better oral health–related quality of life was associated with better sleep quality.

**TABLE 2:** Oral and physical conditions associated with sleep quality.

	Sleep quality		*p*	COR (95% CI)
	Good, *N* (%)	Poor, *N* (%)		
Age				
< 75 years old	340 (61.3)	248 (65.3)	0.214	1
≥ 75 years old	215 (38.7)	132 (34.7)		0.84 (0.64, 1.10)
Gender				
Men	179 (32.2)	96 (25.3)	0.022	1
Women	377 (67.8)	284 (74.7)		1.40 (1.05, 1.88)
Education level				
Low	245 (44.1)	164 (43.2)	0.466	1
Medium	184 (33.1)	139 (36.6)		1.13 (0.84, 1.52)
High	127 (22.8)	77 (20.3)		1.25 (0.87, 1.78)
BMI				
Normal	309 (60.5)	228 (63.2)	0.720	1
Overweight	168 (32.9)	110 (30.5)		1.13 (0.84, 1.52)
Obesity	34 (6.7)	23 (6.4)		1.09 (0.63, 1.92)
Physical condition				
Sarcopenia			0.682	
No	542 (97.5)	372 (97.9)		1
Yes	14 (2.5)	8 (2.1)		1.20 (0.51, 3.03)
Physical frailty				
Robust	464 (84.4)	299 (81.0)	0.187	1
Pre‐frailty to frailty	86 (15.6)	70 (19.0)		1.26 (0.89, 1.79)
Comorbidity				
Non	264 (47.5)	173 (45.5)	0.769	1
One	211 (38.0)	146 (38.4)		1.06 (0.79, 1.40)
More than one	81 (14.6)	61 (16.1)		1.15 (0.78, 1.68)
Oral frailty (0, 1–2, > 2)				
No	37 (6.7)	18 (4.7)	< 0.001	1
Pre‐frailty	426 (76.9)	260 (68.4)		1.25 (0.71, 2.30)
Frailty	91 (16.4)	102 (26.8)		2.30 (1.24, 4.40)
*p*‐for trend			< 0.001	
Tongue coating	4.37 ± 3.3	4.37 ± 3.2	0.985	
Chewing function				
Edentulous				
No	501 (90.1)	357 (94.0)	0.037	1
Yes	55 (9.9)	23 (6.1)		0.59 (0.35, 0.96)
Number of nature teeth				
≥ 20 teeth	331 (59.5)	240 (42.0)	0.264	1
< 20 teeth	225 (40.5)	140 (38.4)		0.86 (0.65, 1.12)
Mobility (level III)				
No	484 (96.6)	334 (93.6)	0.037	1
Yes	17 (3.4)	23 (6.4)		1.96 (1.04, 3.78)
Masticatory performance				
Good/moderate	399 (73.1)	288 (76.8)	0.202	1
Poor	147 (26.9)	87 (23.2)		0.82 (0.60, 1.11)
Swallowing function				
Oral DDK				
TA (time/s)			0.722	
≥ 6 times/s	429 (77.4)	298 (78.4)		1
< 6 times/s	125 (22.6)	82 (21.6)		0.95 (0.69, 1.29)
Perceive swallowing difficulty				
No	228 (41.0)	87 (22.9)	< 0.001	1
Suspected	283 (50.9)	237 (62.4)		2.19 (1.63, 2.98)
Yes	45 (8.1)	56 (14.7)		3.26 (2.06, 5.21)
*p*‐for trend			< 0.001	
Dry mouth				
< 10	527 (94.8)	329 (86.6)	< 0.001	1
≥ 10	29 (5.2)	51 (13.4)		2.82 (1.75, 4.50)
GOHAI (mean ± SD)	54.40 ± 6.0	52.39 ± 6.8	< 0.001	

*Note:* Variation in total sample size across the various characteristics is due to missing data. Educational level was classified into three categories: low (illiterate or elementary school), medium (junior high or high school), and high (technical school, college, or above). Oral frailty: dry mouth, chewing ability, number of teeth, swallowing difficulty and ODK‐TA; Geriatric Oral Health Assessment Index, GOHAI; Geriatric Oral Health Assessment Index; DDK, diadochokinesis.

Table 3 presents the results of logistic regression analyses examining the associations between various oral health conditions and sleep quality, adjusted for age, sex, and educational level. Older adults with swallowing difficulty were more likely to report poor sleep quality [Adjusted Odds Ratio (AOR) = 2.69, 95% CI: 1.65–4.40]. A dose–response relationship was observed across stages of swallowing difficulty, with both suspected and confirmed difficulty groups exhibiting significantly poorer sleep quality compared to individuals without swallowing issues (*p* for trend < 0.001). Similarly, dry mouth was significantly associated with poor sleep quality (AOR = 2.06, 95% CI: 1.25–3.45). In contrast, higher General Oral Health Assessment Index (GOHAI) scores were associated with lower odds of poor sleep quality (AOR = 0.97, 95% CI: 0.95–0.99).

**TABLE 3:** Association of oral conditions and sleep quality in logistic regression analysis.

	Sleep quality	
	AOR (95% CI)	*p*
Age		
< 75 years old	1	
≥ 75 years old	0.77 (0.57, 1.04)	0.111
Sex		
Men	1	
Women	0.77 (0.55, 1.06)	0.091
Education level		
Illiterate/elementary school	1	
Junior high/high school	0.84 (0.61, 1.16)	0.284
Above technical school/college	0.90 (0.61, 1.33)	0.606
Edentulous		
No	1	
Yes	0.60 (0.35, 0.99)	0.047
Mobility (level III)		
No	1	
Yes	1.81 (0.93, 3.58)	0.079
Perceive swallowing difficulty		
No	1	
Suspected	1.96 (1.43, 2.68)	< 0.001
Yes	2.69 (1.65, 4.40)	< 0.001
*p* for trend		< 0.001
Dry mouth		
< 10	1	
≥ 10	2.06 (1.25, 3.45)	0.005
GOHAI	0.97 (0.95, 0.99)	0.004

*Note:* Adjusted for age, sex and educational level.

Abbreviations: AOR, adjusted odds ratio; CI, confidence interval; GOHAI, geriatric oral health assessment index.

Table 4 presents the results of the logistic regression analysis examining the combined effects of oral frailty, physical frailty, and sarcopenia on sleep quality. The combined presence of oral frailty and physical frailty was significantly associated with poor sleep quality (AOR = 2.71, 95% CI: 1.22–6.18).

**TABLE 4:** Logistic regression analysis of the combined effects of oral, physical and sarcopenia on sleep quality.

Variable	Variable	Sleep quality		COR (95% CI)	AOR (95% CI)
		Good, *N* (%)	Poor, *N* (%)		
Oral frailty	Physical frailty				
No/pre‐frailty	No	32 (5.8)	17 (4.6)	1	
Pre‐frailty	No	362 (65.9)	212 (57.5)	1.10 (0.6, 2.08)	1.12 (0.61, 2.11)
Frailty	No	69 (12.6)	70 (19.0)	1.91 (0.98, 3.82)	1.96 (1.01, 3.93)
No	Yes	5 (0.9)	1 (0.3)	0.38 (0.02, 2.59)	0.37 (0.02, 2.57)
Pre‐frailty	Yes	59 (10.8)	38 (10.3)	1.21 (0.60, 2.51)	1.20 (0.59, 2.50)
Frailty	Yes	22 (4.0)	31 (8.4)	2.65 (1.20, 6.02)	2.71 (1.22, 6.18)
*p*‐for trend	0.010				
Oral frailty	Sarcopenia				
No	No	37 (6.7)	18 (4.7)	1	1
Pre‐frailty	No	417 (75.3)	257 (67.6)	1.27 (0.72, 2.32)	1.27 (0.72, 2.34)
Frailty	No	86 (15.5)	97 (25.5)	2.32 (1.24, 4.45)	2.39 (1.28, 4.60)
No	Yes	—	—	—	—
Pre‐frailty	Yes	—	—	—	—
Frailty	Yes	14 (2.5)	8 (2.1)	1.17 (0.40, 3.28)	1.25 (0.43, 3.53)
*p*‐for trend	0.043				

*Note:* Adjusted for age, sex and educational level.

## 4 Discussion

This study identified a significant association between OF and sleep quality in older adults. Poor sleep quality was more prevalent among those experiencing swallowing difficulties and dry mouth. A clear dose–response trend was observed, with worsening sleep quality corresponding to increasing severity of oral frailty and swallowing difficulties. Moreover, older adults who were both orally and physically frail exhibited a particularly strong association with poor sleep quality. These findings underscore the link between oral health–related quality of life and sleep, highlighting the critical relationship between oral function and overall well‐being.

Older adults with increasing severity of oral frailty and swallowing difficulties tend to experience poorer sleep quality. Notably, a higher frequency of impaired swallowing reflexes during sleep has been observed in patients with obstructive sleep apnea (OSA), a well‐established risk factor for sleep disorders. Aging significantly influences the relationship between dysphagia and sleep quality. Data from cohort studies indicate that the prevalence of sleep‐disordered breathing and apnea is higher in individuals aged 60 and above compared to those under 60 years [31]. Similarly, another study reported a greater risk of OSA in adults aged 60–80 years compared to those aged 20–29 years [32]. Insufficient tongue muscle strength, a common condition in older adults, also contributes to dysphagia. Recent research has shown that tongue‐stabilising devices used in individuals with OSA can help maintain tongue position during sleep, thereby reducing fatigue and improving sleep quality [33, 34]. These findings suggest that tongue positioning—particularly when muscle tone decreases during sleep—plays a critical role in sleep quality.

The present study observed that dry mouth was associated with sleep quality. Our findings are consistent with those of a previous study that discussed sleep disruptions associated with dry mouth and sleep disturbances [35]. Previous research has demonstrated that dry mouth can influence the quality of life both directly and indirectly via mediating factors such as dysphagia and masticatory performance among older adults [36]. Salivary secretion is regulated by circadian rhythms and decreases at night. At night, there is less stimulation from speaking and chewing, leading to reduced activation of the parasympathetic nerves, and consequently, decreased saliva production. Additionally, the prevalence of dry mouth increases with age, and polypharmacy exacerbates this condition in older adults. Previous studies investigating self‐reported adverse drug events among community‐dwelling older adults have shown that 12% reported dry mouth and adverse drug events are statistically significantly related to poor sleep quality [37].

Better oral health–related quality of life is associated with improved sleep quality. Numerous studies have established a link between sleep quality and both physical and mental health. In this study, the General Oral Health Assessment Index (GOHAI) was used to evaluate quality of life in older adults, focusing on three domains: physical function, psychosocial function, and pain/discomfort. Our findings revealed that poorer psychosocial aspects of the GOHAI—such as limitations in social interactions due to dental health or denture conditions—were associated with reduced sleep quality. Similarly, a recent review [38] reported that compromised dental function and oral pain can negatively impact sleep. Deterioration in oral health status, reduced oral motor skills, and difficulties with chewing, swallowing, and saliva management have been frequently linked to adverse health outcomes, including physical frailty, decreased quality of life, and increased mortality.

The findings of this study indicate that older adults with coexisting oral and physical frailty are significantly more likely to experience poor sleep quality. This dual frailty not only signals a decline in overall health but also highlights the complex interplay between oral health and physical well‐being. Previous research has shown that the combination of oral and physical frailty is associated with a higher hazard ratio for mortality compared to either condition alone [39]. Moreover, reduced skeletal muscle mass and sarcopenia have been identified as independent risk factors for dysphagia [40, 41], suggesting that degenerative processes affecting both oral and systemic muscle groups may occur concurrently. While past research has primarily focused on the impact of physical frailty, oral muscle weakness can similarly impair food intake, leading to malnutrition and further physical deterioration [10]. Given that oral health influences nutritional status and overall vitality, addressing both oral and physical frailty may be essential for improving sleep quality. These findings underscore the importance of integrated care approaches that target both oral function and physical strength to enhance sleep and overall quality of life in community‐dwelling older adults.

This study had certain limitations. First, this study used a cross‐sectional design; therefore, causality between the variables could not be established. Secondly, although tongue pressure is included in the six‐item OF assessment proposed by Tanaka et al. [8], this study did not measure tongue pressure; future studies should consider including this indicator. However, according to the recent international expert consensus on the operational definition of [9], impaired tongue movement, rather than tongue pressure, is considered one of the key components. In addition, as clarified in the Introduction, the criteria for oral frailty differ from those for oral hypofunction, the latter being defined by the Japanese Society of Gerodontology (2016) based on seven oral signs or symptoms. This clarification may help to avoid conceptual overlap and ensure consistency in future research. Third, only one‐third of the participants were male, which may limit the generalizability of the findings to the broader male adult population in southern Taiwan.

## 5 Conclusion

In conclusion, this study highlights a significant association between OF and sleep quality among community‐dwelling adults. These findings indicate that older adults facing challenges such as swallowing difficulties and dry mouth are likely to experience poorer sleep quality. Furthermore, better oral health‐related quality of life correlates with lower odds of poor sleep quality, suggesting that improvements in oral health can positively impact sleep outcomes. The strong association between concurrent oral and physical frailty and sleep quality underscores the need for holistic health assessments that address both domains. Overall, these results advocate for integrated health interventions aimed at enhancing oral health, and consequently, sleep quality, ultimately contributing to the overall well‐being of older adults.

## Author Contributions

**Pei‐Chen Lin** contributed to the conception, data analysis, interpretation, and writing of the original draft of the manuscript. **Ai‐Hua Chang** and **Shin‐Ru Liao** contributed to the data analysis and interpretation. **Koichiro Matsuo**, **Yuji Kabasawa**, and **Ju‐Hui Wu** contributed to the conception and interpretation of the study. **Pei‐Chao Lin** and **Hsiao‐Ling Huang** contributed to the conception, study design, data analysis, interpretation, and manuscript revision. All authors approved the final version of the manuscript. The data are available upon request from the authors.

## Funding

This work was supported by the National Health Research Institute, Taiwan, NHRI‐107AI‐PHCO03181808.

## Consent

This study was approved by the Institutional Review Board of Kaohsiung Medical University Hospital (KMUHIRB‐E(I)‐20170270, KMUHIRB‐E(I)‐20180122). All participants provided written consent before participation.

## Conflicts of Interest

The authors declare no conflicts of interest.

## Data Availability

The data that support the findings of this study are available from the corresponding author upon reasonable request.
